# Identifying Knowledge Gaps Regarding Heat Stroke Among an Adult Sample in Pune, India: A Descriptive Analysis

**DOI:** 10.7759/cureus.82963

**Published:** 2025-04-25

**Authors:** Suresh Kumar Ray, Bhagyashree A Jogdeo, Ningthoujam Sujita Devi, Manisha J Karkar

**Affiliations:** 1 Nursing, Bharati Vidyapeeth (Deemed to be University) College of Nursing, Pune, IND; 2 Community Health, Bharati Vidyapeeth (Deemed to be University) College of Nursing, Pune, IND

**Keywords:** descriptive study, heat, heat stroke, indian adults, knowledge level

## Abstract

Introduction: Heat stroke is a serious health concern that occurs when the body fails to regulate its temperature, often due to extreme heat exposure. Given the rising temperatures and recurring heat waves in India, understanding and preventing heat stroke is vital. The state of Maharashtra, including Pune city, is prone to extreme heat events, making it necessary to evaluate the knowledge of residents regarding this life-threatening condition. Limited studies exist in this region to assess public knowledge on heat stroke, highlighting the importance of this research. The main objective of this study is to assess the level of knowledge regarding heat stroke among adults residing in selected areas of Pune. The study aims to identify gaps in knowledge and understand the relationship between demographic factors and knowledge levels. This information can support public health initiatives aimed at heat stroke prevention.

Material and methods: A non-experimental, descriptive design was employed using a quantitative research approach. The study was conducted in selected areas of Pune. A total of 300 adult participants aged 18 and above were selected using non-probability purposive sampling. The sample size was calculated considering the finite population of the urban slums, and knowledge deficit was identified from previous literature regarding heat stroke. Data collection was carried out using a self-structured questionnaire, which included demographic variables and specific questions on knowledge of heatstroke. The content validity of the tool was established, and the test-retest method was implemented to ascertain the reliability of the tool. The pilot study was conducted to understand the feasibility of the tool on 30 samples. Frequency percentage analysis was undertaken to analyze the data collected pertaining to the knowledge regarding heatstroke.

Results: Demographic analysis showed that 80 (26.5%) participants were in the 38-47-year age group, and 162 (54%) were female. In terms of education, 162 (54%) had completed education up to the 10^th^ grade and above. The majority of participants (101, 33.5%) had a family income of Rs. 20,001-30,000 (Indian rupees (INR)). Religion-wise analysis shows that 131 (43.5%) of participants were Hindu. Data related to knowledge revealed that 159 (53%) of participants had average knowledge regarding heat stroke, 114 (38%) had good knowledge, and 27 (9%) had poor knowledge. The study found no significant association between knowledge levels and demographic variables such as age, gender, education, family income, and religion (p > 0.05).

Conclusion: The findings indicate that most adults in selected areas of Pune have average knowledge of heat stroke, with only a small proportion demonstrating poor knowledge. The lack of a significant association between knowledge and demographic factors suggests that efforts to increase awareness and knowledge should be broad-based rather than targeting specific groups. Public health campaigns should aim to educate all demographic segments on the dangers of heat stroke, symptoms, and preventive measures. Given the threat posed by rising temperatures in Maharashtra, such initiatives could play a crucial role in reducing the health burden of heat-related illnesses.

## Introduction

It is important to understand heat stroke so that we can prevent it and stay safe, especially during the hot summer months. Our body naturally cools itself, primarily through sweating. However, when the weather is extremely hot or when we engage in intense physical activity in the heat, our body may not cool down quickly enough [1]. Some common causes of heat stroke include prolonged exposure to very hot weather, not drinking enough water, engaging in heavy exercise or work in the heat, and wearing clothes that do not allow sweat to evaporate, which can cause the body to overheat. Heat stroke can be very dangerous, so it is important to recognize the symptoms quickly. A body temperature of 40°C is a key sign of heat stroke. The skin may feel very hot but dry to the touch because the body stops sweating. The heart beats faster as the body tries to cool down. A throbbing headache is common during heat stroke, and the person may feel dizzy or have trouble thinking clearly. They may also feel nauseous. In severe cases, the person may faint or lose consciousness. Heat stroke can be prevented by drinking plenty of water, especially in hot weather, and avoiding drinks with caffeine or alcohol, as they can dehydrate. Lightweight, loose-fitting clothing that allows the body to breathe and cool down should be chosen. If signs of heat stroke are observed in an individual, quick action is essential. The person should be moved out of the sun and into a cool or shaded area. Any available means, such as cool water, fans, or ice packs, should be used to lower their body temperature. Water should be given to them to drink, ensuring it is consumed slowly. Heat stroke is a medical emergency, so it is important to seek medical help immediately [2].

Studying heat stroke is crucial because it affects people's health and safety, especially during extremely hot and humid weather. First, understanding heat stroke helps us determine how common it is, where it occurs most frequently, and what increases the risk of developing it. By analyzing data on heat-related illnesses and deaths, researchers can identify the most vulnerable groups, the most hazardous locations, and the key factors contributing to heat stroke. This information enables public health agencies, policymakers, and healthcare providers to develop effective strategies for protecting people during heatwaves [3-5]. Second, understanding how the body reacts to extreme heat is essential for preventing heat stroke. Learning how our body regulates temperature and adapts to hot environments helps in developing guidelines for managing heat exposure and preventing heat-related illnesses. Research can also lead to improved methods for diagnosing and treating heat stroke early, which can save lives. Finally, studying heat stroke provides insight into why certain groups of people are more affected by heat than others. Factors such as income, access to healthcare, living conditions, and environmental influences can impact a person's risk of developing heat stroke. By examining these differences, researchers can advocate for solutions that protect those most at risk, ensuring that everyone has an equal opportunity to stay safe during extreme heat [6].

## Materials and methods

Ethical considerations

This was approved by the Institutional Research and Recommendation Committee of Bharati Vidyapeeth (Deemed to be University) College of Nursing, Pune, India (approval letter number: BV(DU)CON/IRRC/58). Written informed consent was taken from the selected participants. 

Research design

A non-experimental, descriptive design was employed using a quantitative research approach [7-8].

Setting

The study was conducted in selected urban slums. In the selected slums, the people were from low socioeconomic backgrounds and less educated. Urban slum residents are mostly migrants from rural areas who came to Pune for the sake of earning a living and settled in slums.

Participants

A total of 300 adult participants aged 18 and above were selected using non-probability purposive sampling. The samples were approached by house-to-house visits, and information was collected after taking written informed consent from the participants.

Data collection tool and data collection

Data collection was carried out using a self-structured questionnaire, which included demographic variables and specific questions (24 questions) on knowledge of heat stroke (Table 1). Content validity of the tool was ensured by expert review, and reliability was established through a test-retest method with a Karl-Pearson correlation coefficient (r = +0.7468). A pilot study was conducted with 30 participants to evaluate the feasibility and accuracy of the data collection process. The pilot study was conducted in a similar setting to an urban slum. The pilot study concluded that 15 (50%) of the adults had average knowledge, nine (30%) had good knowledge, and six (20%) had poor knowledge. It also concluded that an actual study is feasible to be conducted.

**Table 1:** The questionnaire distributed to participants to check their knowledge regarding heat stroke

DATA COLLECTION: SELF-STRUCTURED QUESTIONNAIRE							
Instructions:							
Please give the following information correctly							
Place a tick mark on the appropriate answer							
Kindly don’t miss any item							
Information corrected will be strictly confidential							
Section I: Demographic Characteristics of Samples							
1.	Code						
2.	Age						
	A.						18 – 27 years
	B.						28 – 37 years
	C.						38 – 47 years
	D.						48 – 60 years
3.	Gender						
	A.					Male	
	B.					Female	
	C.					Transgender	
4.	Religion						
	A.				Hindu		
	B.				Muslim		
	C.				Christian		
	D.				Other (specify)		
5.	Educational status						
	A.	Primary					
	B.	Secondary					
	C.	10^th^ and above					
6.	Family income (Indian rupees (INR)						
	A.		Less than Rs. 10000				
	B.			Rs. 10001 to 20000			
	C.			Rs. 20001 to 30000			
	D			More than Rs. 30000			
Section 2: Structured Knowledge Questionnaire regarding Heat Stroke							
1	What is the primary cause of heat stroke?						
	a			Excessive physical exertion			
	b			Prolonged exposure to high temperatures			
	c			Dehydration			
	d			All of the above			
2	Which age group is most susceptible to heat stroke?						
	a			Children			
	b			Young adults			
	c			Elderly adults			
	d			Teenagers			
3	What is the recommended amount of water intake during hot weather to prevent heat stroke?						
	a			1-2 glasses per day			
	b			4-6 glasses per day			
	c			8-10 glasses per day			
	d			It depends on the individual factors			
4	Which of the following activities increases the risk of heat stroke?						
	a			Swimming			
	b			Sitting indoor			
	c			Running a marathon			
	d			Watching TV			
5	What is the first aid treatment for someone experiencing heat stroke?						
	a			Apply ice packs to the body			
	b			Move them to a cooler place and fan them			
	c			Give them warm fluids to drink			
	d			None of the above			
6	What body temperature indicates a heat stroke emergency?						
	a			99°F (37.2°C)			
	b			102°F (38.9°C)			
	c			104°F (40°C)			
	d			98.6°F (37°C)			
7	Which of the following conditions can worsen the risk of heat stroke?						
	a			Obesity			
	b			Hypertension			
	c			Diabetes			
	d			All of the above			
8	What is the most common cause of death in heat stroke cases?						
	a			Dehydration			
	b			Organ failure			
	c			Cardiac arrest			
	d			Heat exhaustion			
9	Which body organ is particularly vulnerable to damage during heat stroke?						
	a			Liver			
	b			Kidneys			
	c			Lungs			
	d			Stomach			
10	What is the best way to prevent heat stroke during outdoor activities?						
	a			Wear heavy clothing			
	b			Stay indoors during peak heat hours			
	c			Take frequent breaks in the shade			
	d			Drink hot beverages			
11	Which of the following factors can increase the risk of heat stroke?						
	a			Taking certain medications			
	b			Living in a cold climate			
	c			Being well-hydrated			
	d			Having a high body mass index (BMI)			
12	How does humidity affect the risk of heat stroke?						
	a			High humidity increases the risk			
	b			Low humidity increases the risk			
	c			Humidity has no impact on heat stroke risk			
	d			It depends on individual tolerance			
13	What is the recommended clothing for preventing heat stroke during outdoor activities?						
	a			Tight-fitting clothes			
	b			Dark- coloured clothes			
	c			Loose, light- coloured clothes			
	d			Multiple layers of clothing			
14	Which of the following beverages is best for preventing dehydration and heat stroke?						
	a			Alcohol			
	b			Soda			
	c			Water			
	d			Coffee			
15	What is the best time of day for outdoor activities to avoid heat stroke?						
	a			Early morning			
	b			Noon			
	c			Late afternoon			
	d			Evening			
16	What role does sunscreen play in preventing heat stroke?						
	a			It helps regulate body temperature			
	b			It prevents dehydration			
	c			It protects against sunburn, reducing heat absorption			
	d			It has no effect on heat stroke prevention			
17	How does the body typically cool itself down during hot weather?						
	a			Shivering			
	b			Sweating			
	c			Increased heart rate			
	d			Constricting blood vessels			
18	What is the main difference between heat exhaustion and heat stroke?						
	a			Heat stroke has no symptoms			
	b			Heat exhaustion is less severe than heat stroke			
	c			Heat stroke causes shivering			
	d			Heat exhaustion does not require medical attention			
19	What is the recommended action if you suspect someone is experiencing heat stroke?						
	a			Give them a blanket to warm up			
	b			Encourage them to exercise vigorously			
	c			Call emergency services and provide first aid			
	d			Ignore the situation and continue with your own activities			
20	Which body system does heat stroke primarily affect?						
	a			Respiratory system			
	b			Nervous system			
	c			Muscular system			
	d			Digestive system			
21	What is the most effective way to measure body temperature during heat stroke?						
	a			Touching the forehead			
	b			Using a rectal thermometer			
	c			Using an infrared thermometer			
	d			Using a mercury thermometer			
22	What is the minimum duration of exposure to high temperatures required to develop heat stroke?						
	a			15 minutes			
	b			30 minutes			
	c			1 hour			
	d			2 hours			
23	Which of the following activities should be avoided during a heat wave to prevent heat stroke?						
	a			Exercising outdoors			
	b			Swimming			
	c			Cooking in the kitchen			
	d			Taking a cold shower			
24	What is the main difference between heat stroke and sunstroke?						
	a			Heat stroke is caused by dehydration, while sunstroke is caused by sun exposure			
	b			Heat stroke is more severe than sunstroke			
	c			There is no difference between the two terms			
	d			Sunstroke only affects the skin, while heat stroke affects the entire body			

Analysis

The collected data were processed further for frequency and percentage distribution. A descriptive analysis is used to identify the level of knowledge among urban slums. The chi-square association test was used to identify the association between the level of knowledge and selected demographic variables. The analysis was done through Microsoft Excel (Microsoft Corp., Redmond, WA).

## Results

Analysis of data related to demographic variables

**Figure 1:**
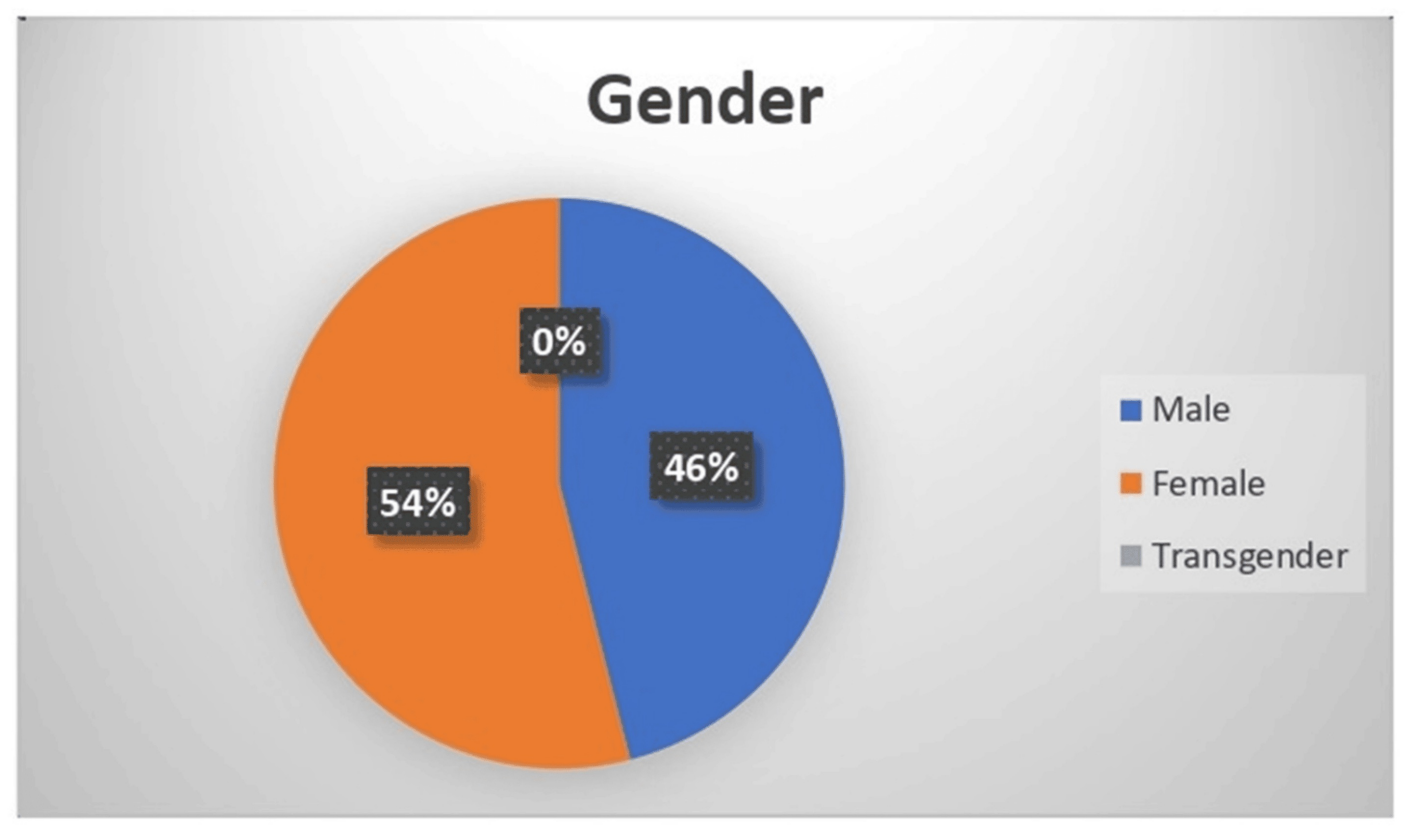
Pie diagram showing gender-wise distribution of the sample (N=300)

**Figure 2:**
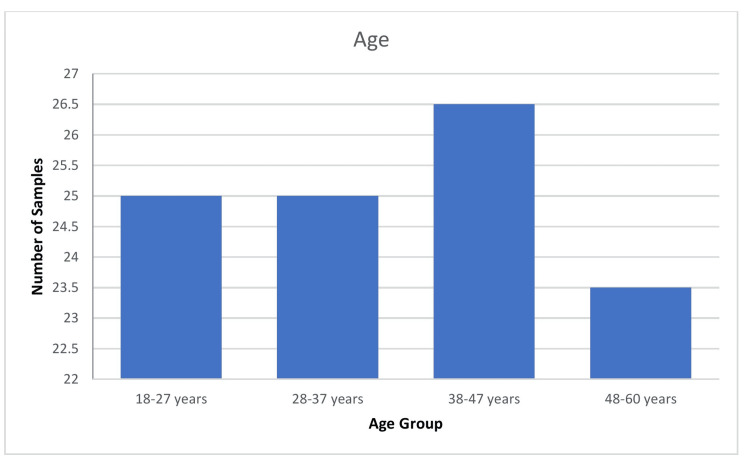
Bar diagram showing age-wise distribution of the sample (N=300)

**Figure 3:**
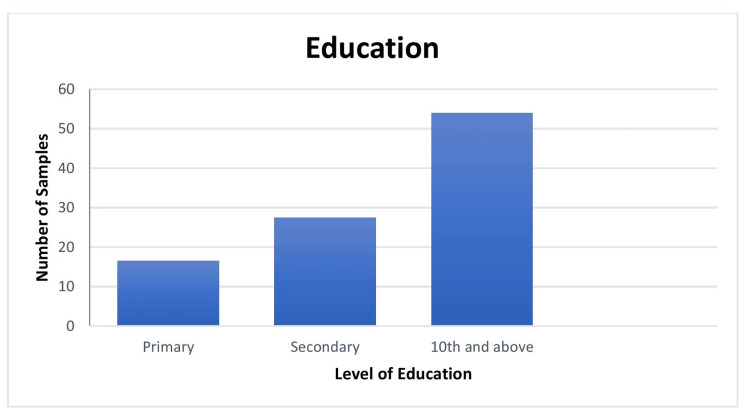
Bar diagram showing the distribution of the sample as per the level of education (N=300)

**Figure 4:**
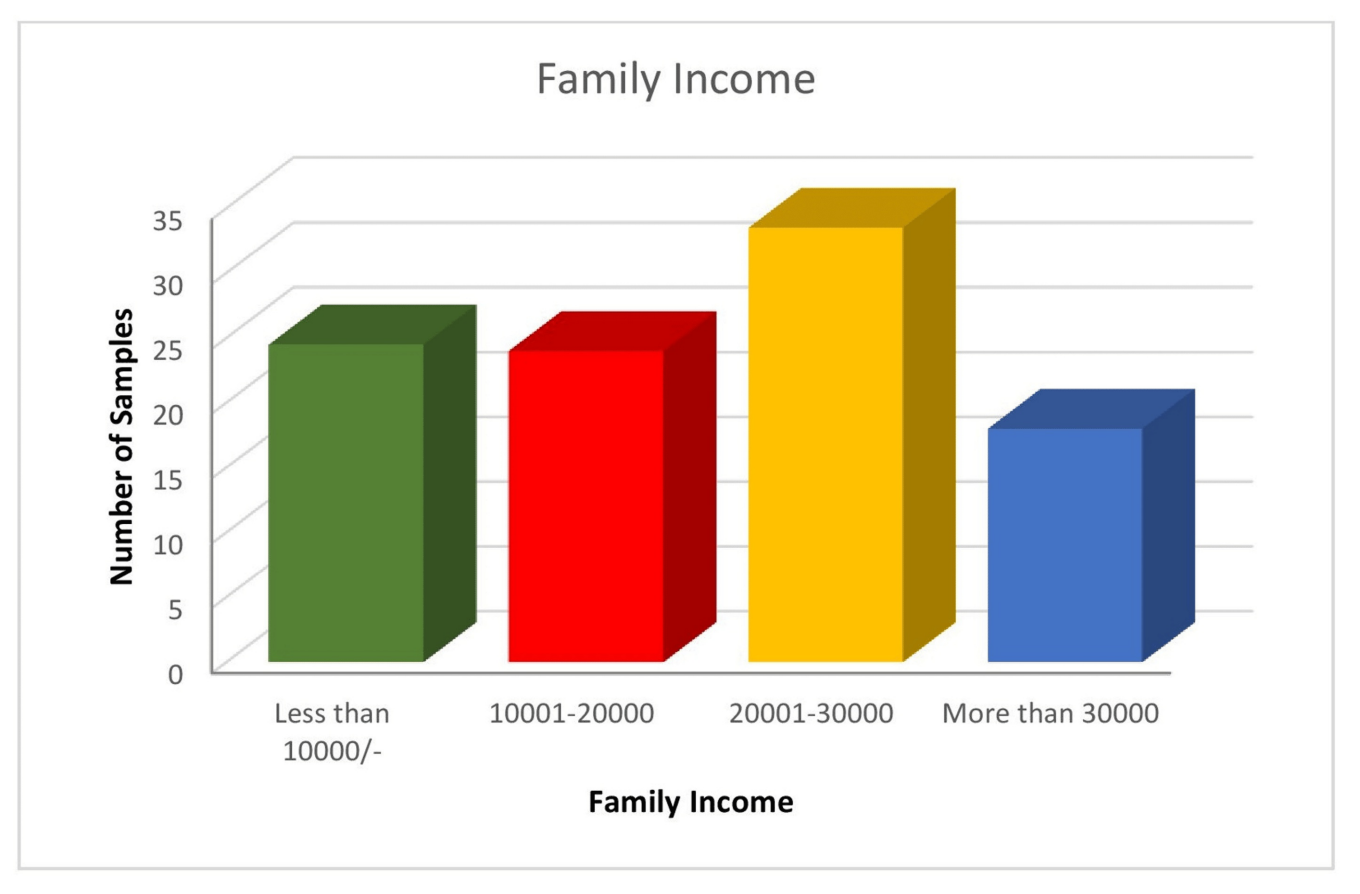
Bar diagram showing the distribution of the sample as per family income (N=300) Income is denoted in Indian rupees (INR).

**Figure 5:**
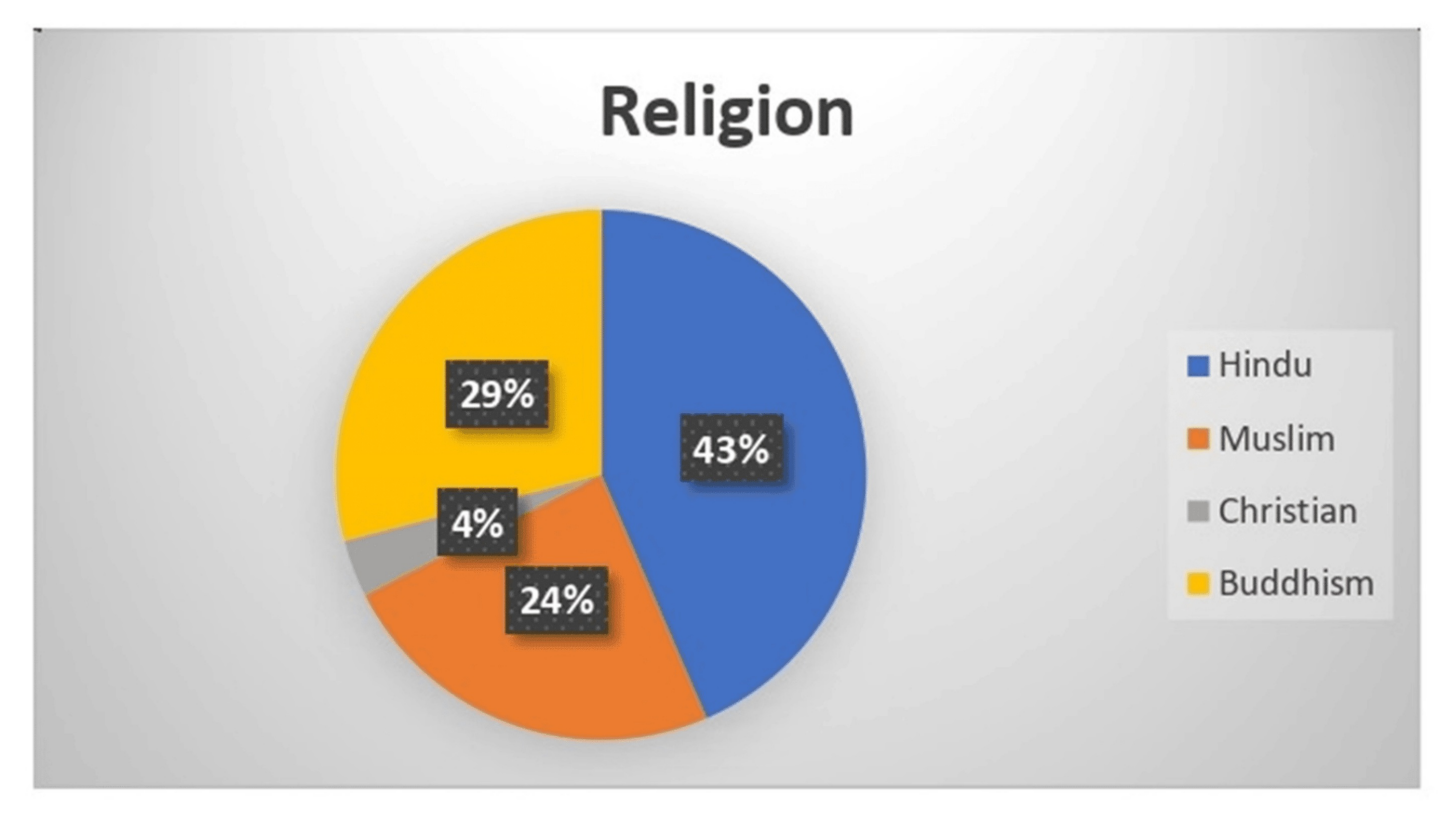
Pie diagram showing the distribution of the sample as per religion (n=300)

The demographic distribution of 300 samples is summarized as follows. Age-wise distribution shows that the majority (80,26.5%) of participants were in the 38-47-year age group. Gender-wise distribution reveals that 162 (54%) participants were female. Regarding education, the majority (162, 54%) of participants had an education level of 10^th^ grade and above. Family income distribution indicates that 101 (33.5%) of participants had a family income between Rs. 20,001 and Rs. 30,000 (Indian rupees (INR)). Religion-wise analysis shows that 131 (43.5%) of participants were Hindu. This comprehensive demographic data provides insight into the socioeconomic and educational background of the sample population (Figures 1-5).

Level of knowledge

**Figure 6:**
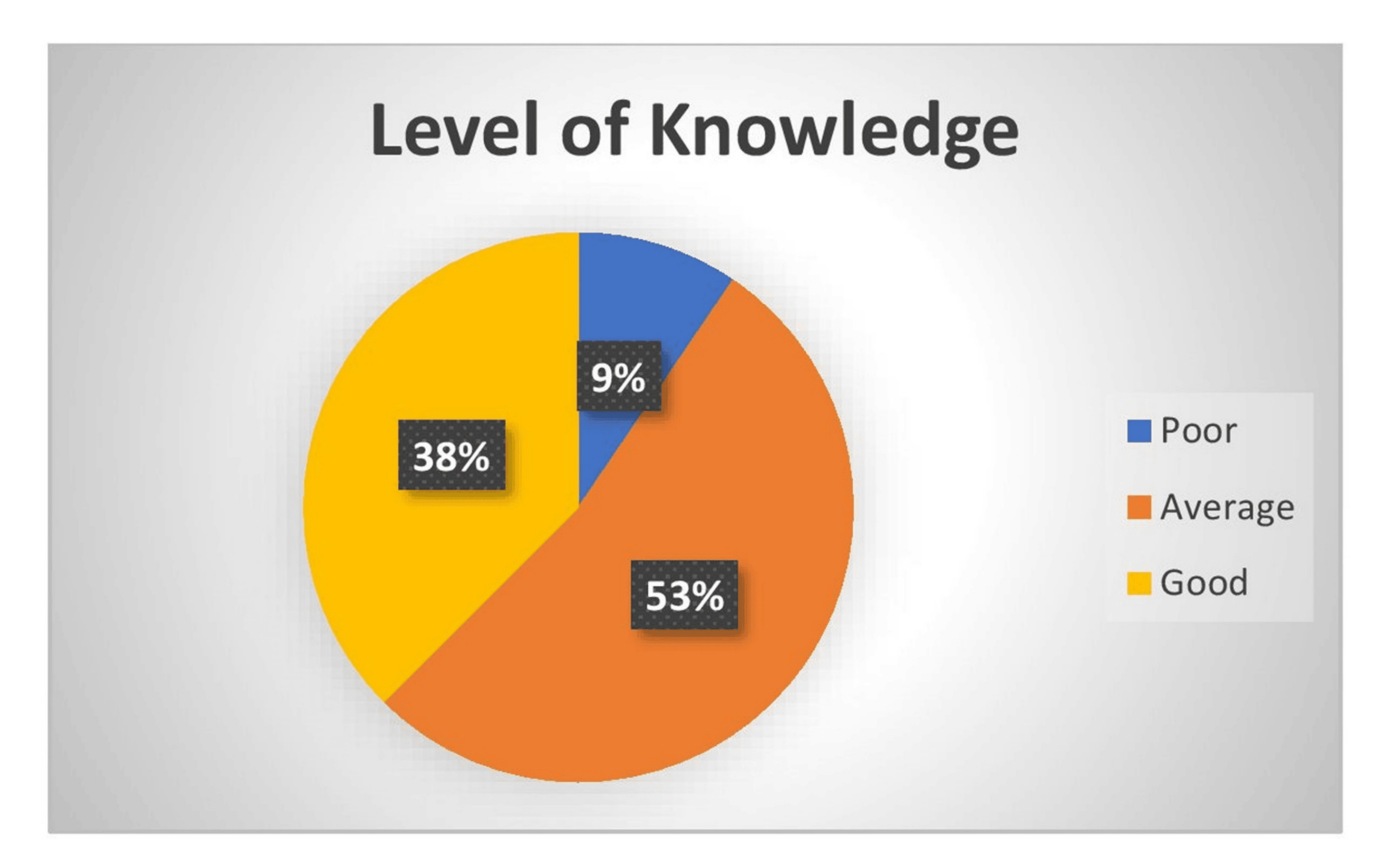
Pie diagram showing the sample's level of knowledge (in percentages) regarding heat stroke

Among the sample, 159 (53%) had average knowledge, 114 (38%) of the samples had good knowledge, and 27 (9%) of the samples had poor knowledge regarding heat stroke (Figure 6).

Association between the level of knowledge and selected demographic variables

There was no association with the knowledge score of adults regarding heat stroke with selected demographic variables like age, gender, education, family income, and religion, since the p-value was more than 0.05 level of significance (Table 2).

**Table 2:** Analysis of association between findings of knowledge and selected demographic variables

Demographic variable	Poor knowledge	Average knowledge	Good knowledge	DF	Chi-square statistics value	Chi-square table value	P-value	Remarks
Age								
18 - 27 years	4	24	22	6	1.446	12.59	0.051	NA
28 - 37 years	3	31	16					
38 - 47 years	4	28	15					
48 – 60 years	8	23	22					
Gender								
Male	9	49	35	4	0.009	3.84	0.9244	NA
Female	10	57	41					
Transgender	0	0	0					
Education								
Primary	10	57	41	4	0.072	3.84	0.7884	NA
Secondary	5	29	21					
10^th^ grade and above	4	20	14					
Family Income (Indian rupees (INR))								
Less than Rs. 10,000	4	26	19	6	1.697	12.59	0.1927	NA
Rs. 10,000 - Rs. 20,000	6	36	25					
Rs. 20,001 - Rs. 30,000	6	25	18					
More than Rs. 30,000	3	19	17					
Religion								
Hindu	8	46	33	6	0.44	12.59	0.5071	NA
Muslim	4	25	18					
Christian	1	4	2					
Buddhism	6	31	22					

## Discussion

Researchers employed a quantitative research approach and chose a non-experimental, descriptive design to gauge participants' awareness and understanding of heat stroke. A total of 300 adults from specific areas of Pune were selected using non-probability purposive sampling. The structured questionnaire used in this study collected data on participants' knowledge and their demographic profiles, including age, gender, education, family income, and religion.

The results showed that most participants had an average level of knowledge regarding heat stroke. Specifically, 159 (53%) of the participants demonstrated average knowledge, 114 (38%) had good knowledge, and 27 (9%) exhibited poor knowledge. The analysis also indicated that the p-values for these associations were greater than 0.05.

This study underscores the need for public health initiatives to improve heat stroke awareness and preventive practices among adults. Given the high temperatures experienced in Pune, understanding heat stroke symptoms, prevention strategies, and management is crucial for community health and safety. The lack of significant association between demographic factors and knowledge suggests that awareness campaigns should be comprehensive and target all sections of society equally, irrespective of socioeconomic status or background [9-10].

The findings also point to the need for additional research to explore potential barriers to acquiring knowledge about heat stroke and to develop more effective educational interventions. The study contributes to a better understanding of how community awareness levels can be enhanced to prevent heat-related illnesses and reduce their impact on the population. Further efforts in promoting accessible and accurate information through public health programs and community outreach could play an important role in equipping individuals with the necessary knowledge to protect themselves during extreme heat conditions.

A similar kind of finding was observed in a study carried out among farmers on their knowledge of heat stroke. This descriptive study was conducted among 42 farmers. A structured questionnaire was used to assess the participants' knowledge of heat stroke and its prevention. Data were analyzed using descriptive statistics, including frequency distributions and percentages. The study found that 61.90% of the sample had moderate knowledge, 35.71% had adequate knowledge, and 2.385% had inadequate knowledge regarding heatstroke. The future study should enhance and promote the knowledge and prevention of heatstroke among farmers worldwide [4].

The limitation of the study is that the responses towards the knowledge regarding heat stroke are purely based on the responses of the respondents. Secondly, the findings of the study are limited to the selected settings of urban slums.

## Conclusions

This study highlights the critical need to understand heat stroke, a life-threatening condition exacerbated by hot weather and insufficient hydration. The research provided a clear picture of awareness levels and demographic influences. The initial assumption that the adults may have some knowledge regarding heat stroke was proven to be the same, as the majority of them had average knowledge regarding heat stroke. The grey areas of knowledge were risk factors and immediate management of heat stroke. Distribution of knowledge levels did not show a statistically significant association. The study underscores a need for targeted awareness campaigns to improve understanding and preventive measures against heat stroke, especially in vulnerable populations.
